# Development and Characterization of Topical Gels Containing Lipid Nanosystems Loaded with *Echinacea purpurea*

**DOI:** 10.3390/gels11100801

**Published:** 2025-10-05

**Authors:** Ramona-Daniela Pavaloiu, Georgeta Neagu, Adrian Albulescu, Mihaela Deaconu, Anton-Liviu Petrica, Corina Bubueanu, Fawzia Sha’at

**Affiliations:** 1 National Institute for Chemical-Pharmaceutical Research and Development, ICCF, 031299 Bucharest, Romania; pavaloiu.daniella@gmail.com (R.-D.P.); getabios@yahoo.com (G.N.); adrian.albulescu@virology.ro (A.A.); corina.bubueanu@yahoo.com (C.B.); 2 Department of Molecular Virology, Stefan S. Nicolau Institute of Virology, 030304 Bucharest, Romania; 3 Faculty of Chemical Engineering and Biotechnologies, University Politehnica of Bucharest, 011061 Bucharest, Romania; mihaela_deaconu@yahoo.com; 4 Faculty of Business and Tourism, Bucharest University of Economic Studies, 010374 Bucharest, Romania; petricaanton20@stud.ase.ro

**Keywords:** lipid nanosystems, gel, plant extract, *Echinacea purpurea*

## Abstract

This study explores an innovative delivery strategy for the management of skin conditions: lipid nanosystems incorporated into a gel matrix. *Echinacea purpurea* extract, known for its antibacterial, antioxidant, and wound-healing properties, was encapsulated into lipid-based nanosystems and subsequently incorporated into Carbopol-based gel. The extract, rich in chicoric and caftaric acids, exhibited strong antioxidant activity (IC_50_ = 56.9 µg/mL). The resulting nanosystems showed nanometric size (about 200 nm), high entrapment efficiency (63.10–75.15%), and excellent short-term stability. Superior biocompatibility of the nanosystems, compared to the free extract, was demonstrated using an MTS assay on L-929 fibroblasts. Moreover, the cytoprotective potential of the lipid carriers was evident, as pre-treatment significantly increased cell viability under H_2_O_2_-induced oxidative stress. These findings suggest that lipid-based encapsulation enhances the therapeutic profile of *E. purpurea.* The optimal lipid formulation was incorporated into a Carbopol-based gel, which demonstrated an appropriate pH (5.15 ± 0.75), favorable textural properties, sustained polyphenol release, and overall good stability. This research highlights the potential of plant-derived bioactives in the development of dermatocosmetic products, aligning with current trends in eco-conscious and sustainable skincare.

## 1. Introduction

The skin is the largest organ of the human body [1] and serves as a multifunctional barrier that protects against environmental aggressors while regulating hydration, thermoregulation, and immune responses. Maintaining skin integrity is a primary focus of both clinical dermatology, which aims to treat and prevent pathological conditions, and the cosmetics industry, which prioritizes skin appearance, comfort, and consumer satisfaction [1,2,3]. In recent years, there has been a growing interest in multifunctional skincare strategies that combine protection, regeneration, and aesthetic benefits in a single formulation. Examples include antioxidant-enriched emulsions that neutralize free radicals; barrier-strengthening lipid formulations that restore stratum corneum integrity; and regenerative gels containing bioactive peptides or botanical extracts.

Topical formulations enriched with natural bioactives have emerged as promising solutions for skin management. Plant-derived compounds, including polyphenols, flavonoids, and selected essential oils (e.g., tea tree, lavender), are known for their antioxidant, anti-inflammatory, and regenerative effects, which help maintain skin homeostasis and mitigate oxidative stress and inflammation [4,5,6]. They can also support collagen synthesis and maintain skin elasticity, thereby contributing to healthier, more resilient skin. However, their practical application is limited by variable safety and efficacy across different plant species, as well as challenges related to chemical instability, poor solubility, and restricted penetration through the stratum corneum [7].

Lipid nanosystems have emerged as versatile nanocarriers to address these limitations. Liposomes, composed of phospholipid bilayers with aqueous cores, can encapsulate hydrophilic and lipophilic molecules, shielding them from degradation and enhancing delivery to the stratum corneum and upper epidermis. Transferosomes, or ultra-deformable vesicles containing edge activators, adapt their morphology to traverse intercellular lipid pathways and may reach deeper layers of the viable epidermis; however, consistent evidence of true dermal penetration remains debated [8,9]. Solid lipid nanoparticles (SLNs) and nanostructured lipid carriers (NLCs) combine solid matrices with biocompatible lipids, enabling controlled release and sustained bioavailability [10,11]. When incorporated into gel formulations, these lipid carriers not only enhance solubility and stability but also provide a hydrating, non-greasy, and user-friendly matrix suitable for topical application, thereby improving patient compliance and cosmetic acceptability [12].

Among medicinal plants, *Echinacea purpurea* (L.) Moench has attracted particular attention due to its high content of caffeic acid derivatives, alkamides, and polysaccharides. These compounds exhibit antioxidant, anti-inflammatory, and skin-repairing activities [7,13]. Studies have also shown that *E. purpurea* extracts can modulate inflammatory mediators and growth factors involved in tissue repair, supporting skin homeostasis and regeneration [9,14]. Despite these advantages, direct application of *E. purpurea* extracts is limited by poor stability, susceptibility to oxidation, and restricted skin permeability.

Among medicinal plants, *E. purpurea* (L.) Moench has been widely investigated for its caffeic acid derivatives, alkamides, and polysaccharides. These constituents exhibit antioxidant, anti-inflammatory, and tissue-repairing properties [7,13]. Extracts of *E. purpurea* have also been reported to modulate inflammatory mediators and growth factors such as TNF-α, IL-6, and VEGF, which have roles in tissue repair and regeneration [9,14]. However, extract composition may vary depending on cultivation and processing conditions, including soil, altitude, and extraction solvent, which complicates standardization [15]. Furthermore, direct application of *E. purpurea* extracts is restricted by their instability, susceptibility to oxidation, and limited skin permeability.

Encapsulating *E. purpurea* extracts within lipid nanosystems, followed by incorporation into gel matrices, represents a promising strategy to overcome these limitations. Lipid-based nanocarriers, such as liposomes, transferosomes, and SLNs, can protect the bioactive compounds from degradation, enhance their solubility, and facilitate penetration through the skin barrier [16,17,18]. Transferosomes, owing to their deformability, can improve penetration through intercellular lipid channels, although their capacity to achieve consistent dermal delivery requires further investigation. Incorporating these carriers into gels provides additional benefits: hydrating effects that enhance skin comfort, tunable rheology for better adhesion and spreadability, and controlled release for sustained bioactivity [19,20]. For example, Sahu et al. demonstrated the potential of nanostructured lipid carrier-based gels for plant-derived compounds by achieving prolonged transdermal release and enhanced skin interaction of anti-inflammatory agents, as evidenced by in vitro release, ex vivo permeation, and skin retention/dermatokinetic studies [21].

The originality of this study lies in combining the well-documented bioactivity of *E. purpurea* with the technological advantages of lipid nanosystems and gel carriers, producing a formulation that addresses both stability and delivery challenges. Such an approach offers potential applications in daily skincare, wound healing, and the management of oxidative or inflammatory skin conditions, bridging the gap between traditional plant-based remedies and advanced topical therapeutics.

By combining nanotechnology with sustainable, plant-based actives, this work bridges traditional phytotherapy with advanced dermatocosmetic solutions.

In this study, we prepared and characterized lipid-based nanosystems incorporating *E. purpurea* extract and evaluated their potential for topical delivery. The extract was first characterized in terms of polyphenolic composition and antioxidant activity, then incorporated into liposomes and transferosomes, which were assessed in terms of particle size, polydispersity, entrapment efficiency, stability, and release profile. Their cytotoxicity and cytoprotective effects were tested on fibroblast cultures to confirm safety and biological activity, while noting the necessity of future studies in keratinocytes and skin permeation models. Based on the superior performance of liposomes, we further developed Carbopol-based gels containing these nanocarriers, alongside a comparative gel with free extract, and investigated their physicochemical stability, textural properties, antioxidant capacity, and sustained release behavior. Altogether, this integrated approach allowed us to demonstrate that nanosystem-loaded gels represent an effective and biocompatible strategy for delivering *E. purpurea* bioactives in dermatocosmetic applications.

## 2. Results and Discussion

### 2.1. Phytochemical Characterization and Antioxidant Properties of E. purpurea Extract

The hydroalcoholic extract of *E. purpurea* contained a total polyphenol content of 14.7 ± 0.03 mg GAE/g dry extract. HPLC-DAD analysis, based on comparison of retention times and UV–Vis spectra with authentic standards, revealed chicoric and caftaric acids as the main constituents, highlighting the extract’s strong antioxidant potential. The calibration parameters (RT, λmax, regression equation, R^2^) for all reference standards are provided in Supplementary Table S1. Literature reports show a wide variability in total polyphenol content for *E. purpurea* leaf extracts, for example, 2.7 ± 0.2 mg GAE/g of dry extract [22] or 22.3 ± 1.0 mg GAE/g of dry extract [23]. These differences can be explained not only by agro-climatic factors (altitude, rainfall, soil composition) but also by differences in extraction methodology. In the present study, we used a 50% ethanol (*v*/*v*) hydroalcoholic solvent to optimize recovery of caffeic acid derivatives, while other studies employed distinct solvent bases (such as methanol or alternative ethanol concentrations), which are known to significantly influence polyphenol yield. Thus, both environmental conditions and solvent selection contribute to the variability observed among reported values. Similar trends have been documented for other plant species as well [15,24].

**Figure 1:**
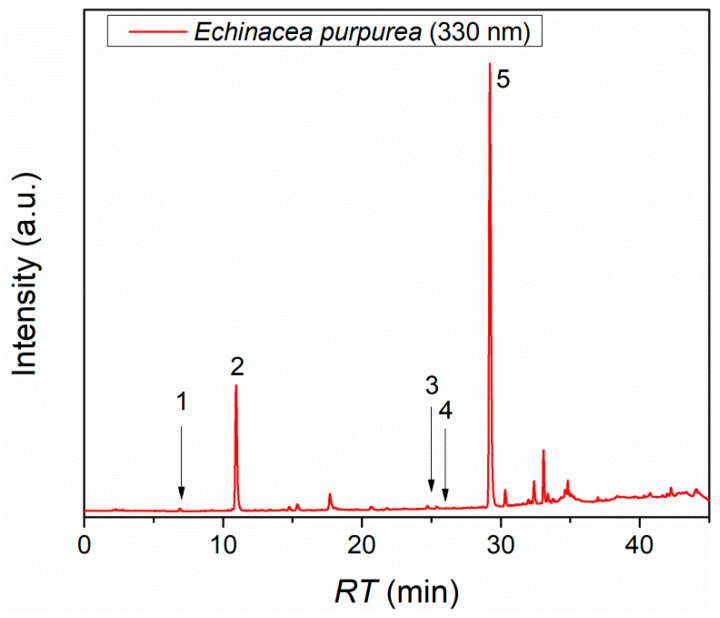
Chromatogram of the *E. purpurea* extract at 330 nm.

Referring to the HPLC-DAD analysis of the *E. purpurea* extract, performed on a C18 reversed-phase column under gradient elution with formic acid–water (A) and acetonitrile–formic acid (B) as mobile phases, nine reference compounds (protocatechuic acid, caftaric acid, vanillic acid, syringic acid, (-)-epicatechin, trans-ferulic acid, ellagic acid dihydrate, rutin hydrate, chicoric acid) were used (Supplementary Table S1). Five polyphenolic compounds were identified and quantified, as presented in Table 1 and the chromatogram (Figure 1). The predominant compounds in *E. purpurea* are chicoric acid, with a concentration of 19.53 ± 0.01 mg compound/g dry extract, and caftaric acid, with a concentration of 15.42 ± 0.02 mg compound/g dry extract, both of which exhibit strong antioxidant, anti-inflammatory, cytomodulatory, and cytostimulatory activities. These values represent the mean ± SD of three independent HPLC injections, and the low SD reflects the high reproducibility of the analytical method.

The *E. purpurea* extract shows a significant ability to scavenge free radicals, with an IC_50_ of 56.9 ± 0.6 µg/mL. This value is at the lower, more favorable end of the range reported in the literature for *E. purpurea* extracts, indicating significant antioxidant potency (lower IC_50_, higher activity; however, direct comparisons should take into account the plant component, solvent, extraction/standardization, and test conditions. Sudeep et al. reported a value of IC_50_ ≈ 106.7 µg/mL for a standardized hydroalcoholic extract of *E. purpurea* enriched to 4% chicoric acid; this is a weaker activity than ours, which is probably due to composition and extraction details [25]. According to Russo et al., methanolic extracts of *E. purpurea* exhibited IC_50_ = 139 ± 0.9 µg/mL, which was once weaker than our extract [26]. However, other studies have reported values of IC_50_ about 15.7 µg/mL for specific ethanolic extracts, indicating a greater antioxidant activity and showing how the choice of solvent, plant part, and phenolic composition can shape the results [27]. Our result is consistent with the range reported for other *E. purpurea* preparations, where DPPH IC_50_ values have been estimated between about 65 and 92 µg/mL [27,28]. Overall, our IC_50_ of 56.9 µg/mL aligns with previously reported data for *E. purpurea*, showing that the extract has strong antioxidant activity. The recovery of caffeic-acid derivatives (caftaric, chlorogenic, and chicoric acids) and other phenolic compounds is influenced by (i) the polarity of the extraction solvent and the optimization of extraction conditions (e.g., hydroalcoholic versus methanol, ultrasound-assisted extraction), and (ii) specific assay parameters such as DPPH concentration, incubation time, and matrix/blank corrections, all of which can affect the observed IC_50_. These factors likely account for the variations reported across different studies [26].

### 2.2. Characterization of Lipid Nanosystems Loaded with E. purpurea

The size, polydispersity index (PDI) and entrapment efficiency (EE) of transferosomes and liposomes loaded with *E. purpurea* extract are presented in Table 2. Empty transferosomes displayed an average particle size of 102.2 ± 1.10 nm with a PDI of 0.40 ± 0.03, while the incorporation of the extract (EP_T) led to a significant increase in particle size (156.3 ± 1.1 nm) and an improvement in homogeneity, as indicated by a lower PDI value (0.08 ± 0.02). For liposomes, the empty nanosystems exhibited an average particle size of 63.2 ± 0.4 nm and a PDI of 0.42 ± 0.02. After loading with *E. purpurea* extract (EP_L), their size increased substantially to 199.1 ± 0.2 nm, while the PDI remained relatively high (0.44 ± 0.01), suggesting a broader size distribution compared with transferosomes. Empty liposomes exhibited a smaller size (63.2 ± 0.4 nm) compared to empty transferosomes (102.2 ± 1.1 nm), maybe due to the intrinsic nature of liposomes, in which bioactive molecules form structured complexes with phospholipid heads, resulting in more compact vesicular assemblies [29]. In contrast, transferosomes, due to the presence of edge activators, form more loosely organized vesicles [30,31]. After loading with *E. purpurea* extract, both nanosystems increased in size (to 156.3 ± 1.1 nm for EP_T and 199.1 ± 0.2 nm for EP_L), consistent with the incorporation of active compounds into the vesicles. Also, loading enhanced the uniformity of transferosomes significantly, as shown by a dramatic decrease in PDI (from 0.40 ± 0.03 to 0.08 ± 0.02), suggesting that the extract reinforces membrane cohesion and promotes uniform vesicle formation. In contrast, liposomes maintained a uniformly broad size distribution (PDI 0.42 ± 0.02– 0.44 ± 0.01) regardless of loading.

The EE for EP_T was 63.10 ± 1.03%, suggesting a great incorporation of polyphenolic compounds within the lipid nanosystems. Liposomes achieved a higher EE (75.15 ± 1.24%) than transferosomes, confirming the strong affinity and complexation between phytochemicals and phospholipids, which promotes better entrapment [32].

In terms of stability, the results shown in Table 2 indicated that the lipid nanosystems containing plant extract remained stable for at least three months, with only minimal loss of phytoconstituents.

### 2.3. Biocompatibility of the E. purpurea Extract and Its Lipid-Based Nanosystems Evaluated by Cytotoxicity Assay

**Figure 2:**
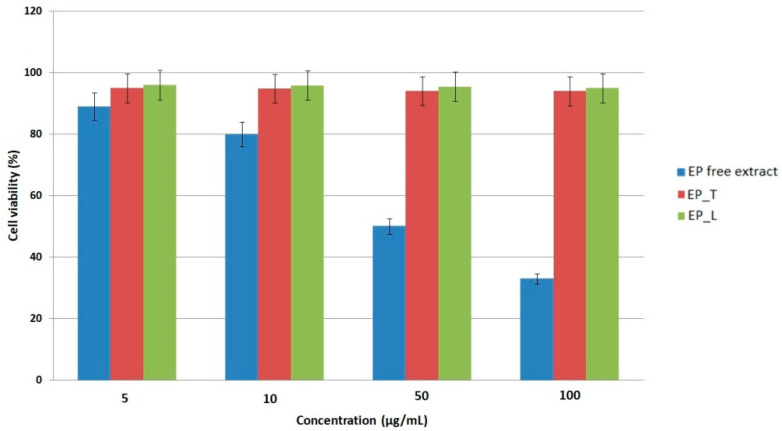
Cytotoxicity profiles of *E. purpurea* extract and its lipid nanosystems (EP_T—transferosomes loaded with *E. purpurea*, EP_L—liposomes loaded with *E. purpurea*. Data are expressed as mean ± SD (*n* = 3).

The cytotoxicity of the *EP* extract and its lipid-based nanosystems was evaluated on L-929 murine fibroblasts using the MTS assay. The free extract showed an IC_50_ value of 64.1 ± 2.9 µg/mL, indicating a moderate decrease in fibroblast viability at higher concentrations (Figure 2). Cells were incubated with the extract or nanosystems for 24 h prior to viability assessment to ensure comparability. At low concentrations (5–10 µg/mL), the extract maintained high cell viability, while higher doses caused a significant decline, reaching only 33% viability at 100 µg/mL, and confirming the concentration-dependent nature of the response. This finding is in line with previous studies demonstrating that polyphenol-rich *Echinacea* extracts may exert dose-dependent cytostatic or cytotoxic effects, largely through redox modulation and the regulation of signaling pathways such as NF-κB and MAPK [33,34]. The biocompatibility profile of the lipid nanosystems, on the other hand, was noticeably superior. Transferosomes and liposomes maintained fibroblasts’ viability at about 94% and 96%, respectively, over a 24 h incubation period. This is significantly higher than the 70% criterion for non-cytotoxic materials specified by ISO 10993-5 [35]. ANOVA analysis confirmed that the difference between free extract and nanosystems was statistically significant (*p* < 0.05). This excellent tolerance implies that the edge activators and phospholipid bilayers used in the nanosystems are intrinsically safe and do not inhibit the proliferation of fibroblasts. Similar outcomes have been reported for various lipid vesicles filled with plant extracts, where nanocarriers enhanced the stability and prolonged release of bioactives while reducing cytotoxicity [36,37,38,39]. The encapsulation of polyphenolic compounds within the lipid bilayers is responsible for the nanosystems’ improved biocompatibility when compared to the free extract. Encapsulation provides a controlled release of bioactive compounds, ensuring a consistent therapeutic effect while preventing cellular stress that could occur from sudden high local concentrations. This protective role of nanosystems has also been widely reported for other phytochemicals and is considered a major benefit for topical use [40]. These results show that transferosomes and liposomes are safe, biocompatible carriers for *E. purpurea* extract. Their ability to maintain fibroblast viability demonstrates their strong potential for use in topical gels targeting infectious skin diseases, where protecting skin cell integrity is essential for effective therapy.

While the use of L929 fibroblasts is appropriate for evaluating cytotoxicity related to dermal matrix integrity and wound-healing processes, it is acknowledged that this model does not fully capture the complexity of skin tissue. As keratinocytes are the predominant cell type in the epidermis and play a central role in barrier function and initial responses to topical applications, future studies will incorporate keratinocyte-based models to provide a more comprehensive assessment of the safety and biocompatibility of E. purpurea nanosystems. Additionally, considering that transferosomes are intended to enhance skin penetration, further investigations involving permeation studies using reconstructed human skin models or ex vivo skin are warranted.

It is important to note that while the MTS assay provided valuable insights into cytotoxicity, this test alone cannot be directly equated with the full spectrum of biocompatibility, which also encompasses hemocompatibility, irritation, sensitization, and long-term tissue compatibility. In this study, cytotoxicity and cytoprotective assays were selected as initial indicators of safety and cellular tolerance, particularly relevant for topical applications. However, comprehensive biocompatibility evaluation according to ISO 10993 standards [35] would require additional investigations, including skin irritation and sensitization models. This represents a limitation of the present study, and additional testing is proposed as a future step to fully validate the suitability of these nanosystem-based gels for clinical dermatocosmetic use.

### 2.4. Enhanced Cytoprotection by E. purpurea Nanosystems Against H_2_O_2_-Induced Oxidative Stress

**Figure 3:**
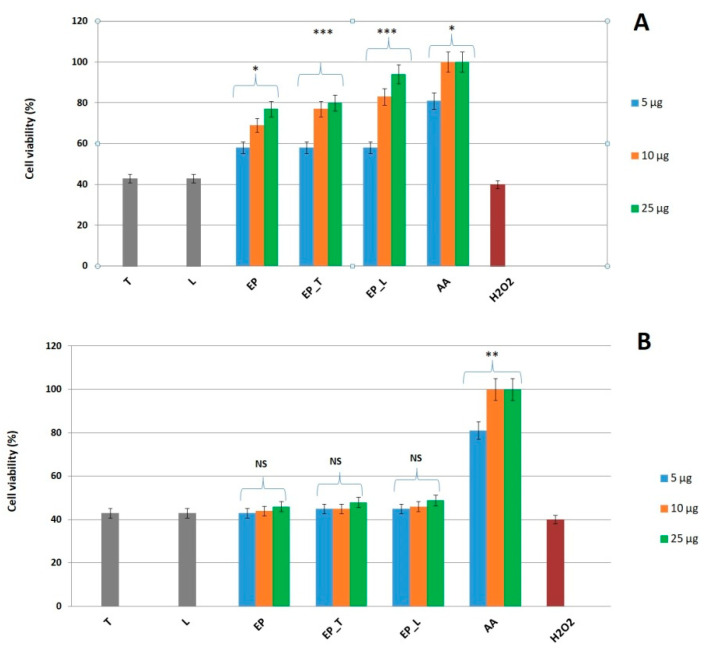
Comparative effect of the free extract and its lipid nanosystems on L-929 cells—pretreatment for 1 h (**A**) or 24 h (**B**) followed by H_2_O_2_ exposure. T—empty transferosomes; L—empty liposomes; EP—E. purpurea free extract; EP_T—transferosomes loaded with E. purpurea; EP_L—liposomes loaded with E. purpurea; AA—ascorbic acid. Statistical analysis performed using ANOVA. Statistical significance is indicated by asterisks (* *p* < 0.05, ** *p* < 0.01, *** *p* < 0.001); NS means not significant. Data are expressed as mean ± SD (*n* = 3).

The cytoprotective activity of the *E. purpurea* extract and its lipid formulations (transferosomes and liposomes) was evaluated in an oxidative stress model using L929 fibroblasts. Exposure to 50 mM H_2_O_2_ reduced fibroblast viability to ~40%, providing a reliable condition for testing protection. Cells were pretreated with the free extract or nanosystems for either 1 h or 24 h, followed by 4 h H_2_O_2_ exposure and viability assessment by MTS assay. Only the 24 h pretreatment significantly improved fibroblast survival, whereas 1 h exposure did not provide protection. This indicates that a longer incubation period is necessary, likely because internalization and cellular responses (e.g., antioxidant enzyme activation) are required for protection. Among the tested samples, *E. purpurea*—loaded transferosomes and liposomes showed stronger protective effects than the free extract, as shown in Figure 3. This is consistent with their superior biocompatibility, as the nanosystems preserved about 94–96% cell viability over 24 h, well above the ISO 10993-5 [35] threshold of 70% for non-cytotoxic materials. The improved performance of nanosystems can be attributed to the encapsulation of polyphenols, which enables gradual release, prevents harmful concentration spikes, and maintains a steady supply of bioactives that support antioxidant defenses and cell survival. In comparison with other plant extracts tested under similar conditions, *E. purpurea* displayed a lower intrinsic antioxidant and protective activity (e.g., compared to *Lycium barbarum* extract) [41]. However, encapsulation markedly enhanced its effect, demonstrating the added value of lipid nanocarriers for extracts with moderate activity. Our findings corroborate earlier studies on lipid vesicles containing phytochemicals. For instance, nanocarriers with *Rosa canina* extract, genistein or thymoquinone considerably improved cell survival while mitigating oxidative or chemical stress compared to the free compounds [35,36,42]. Likewise, quercetin niosomes exhibited improved delivery and antioxidant activity on the skin [39]. More recent reviews have noted lipid-based nanocarriers as having particular efficacy in stabilizing and extending the antioxidant activity of phenolic compounds [38]. For *E. purpurea*, in particular, there have been other studies that have connected its protective and wound-healing properties to the modulation of NF-κB, MAPK, TGF-β, and VEGF signaling pathways [14]. This aligns with our observation that only long-term (24 h) pretreatment was protective, suggesting that *E. purpurea*’s cytoprotective effect depends not only on direct radical scavenging but also on modulation of intracellular signaling and gene expression.

A limitation of the present study is that intracellular polyphenol concentrations and the activation of antioxidant signaling pathways were not directly measured at different pretreatment times. Consequently, the absence of protection following 1 h pretreatment is likely attributable to insufficient internalization of nanosystems and/or incomplete activation of intracellular antioxidant responses, but this mechanism has not been directly confirmed. Future investigations involving quantitative assessment of intracellular polyphenol uptake and analysis of signaling pathway activation (e.g., NRF2, MAPK, NF-κB) are required to clarify the kinetics of nanosystem-mediated cytoprotection.

In conclusion, transferosomes and liposomes loaded with *E. purpurea* showed stronger cytoprotection against oxidative stress than the free extract. These nanosystems, along with their excellent safety profile, represent promising carriers for topical applications targeting infectious skin diseases, where oxidative stress is a major contributor to tissue damage and impaired healing.

### 2.5. Physicochemical and Textural Stability of Gels

The components of the gel formulations were selected to provide both structural stability and functional benefits. Carbopol 940 acted as the gelling agent, creating a stable matrix suitable for topical delivery. Glycerin served as a humectant, promoting uniform mixing of the components and contributing to skin hydration. Sodium hydroxide had a dual role: inducing gelation and adjusting the pH to values compatible with skin application. The final pH range after adjustment was 5.1–5.3, which lies within the physiological skin range. Liposomes containing *E. purpurea* were chosen for the gel formulation because they showed better entrapment efficiency, promoted higher cell proliferation, and exhibited lower cytotoxicity than the transferosomes, thereby improving the delivery of bioactive compounds. *Eucalyptus* essential oil, included at a 0.1% concentration, not only imparted a refreshing aroma but also contributed functional benefits, including antimicrobial, anti-inflammatory, and soothing effects, making it a valuable component in the formulation. Vitamin E, a fat-soluble antioxidant, played a key role in protecting sensitive ingredients like polyphenols and essential oils from oxidative degradation during storage and use. This helps keep the formulation stable, prevents it from rancidity, and extends its overall shelf life. Additionally, vitamin E brings added benefits to the skin, thanks to its soothing, anti-inflammatory, and regenerative properties, making it a valuable ingredient for both product stability and skin health.

**Figure 4:**
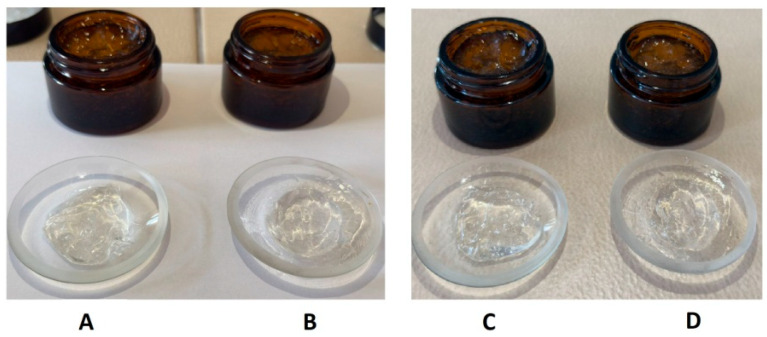
Visual comparison of gel formulations containing *E. purpurea* extract: freshly prepared—(**A**) Gel_EP, (**B**) Gel_EP_L versus after 2 months at 4 °C—(**C**) Gel_EP, (**D**) Gel_EP_L.

The physicochemical properties of gels were assessed initially (after 1 day) and following a 2-month storage period at 4 °C (Table 3 and Figure 4). Both formulations maintained a homogeneous, visually transparent appearance with a specific aromatic odor throughout the evaluation period. The aromatic odor is attributed to the extract and volatile constituents of eucalyptus oil, which remained stable during the storage period. No signs of phase separation, sedimentation, or texture alteration were observed after two months, indicating excellent physical stability of both gels. The pH values of the formulations remained relatively stable over time, with only minor increases observed (Table 3). These values fall within the skin-compatible pH range (approximately 4.5–6.0) [43], supporting their suitability for topical application.

The texture profile analysis (TPA) provided valuable insight into the structural stability of the two gel formulations during storage. Textural properties showed some differences after 1 day. The gel containing liposomes loaded with *E. purpurea* exhibited higher firmness (0.50 ± 0.01 N) compared to the gel with free *E. purpurea* (0.43 ± 0.02 N), suggesting a denser and more resistant structure. Similarly, springiness was greater in the liposome-loaded gel (0.85 ± 0.02) than in the free *E. purpurea* gel (0.74 ± 0.02), indicating enhanced elasticity and recovery after deformation. Cohesiveness values were nearly identical (0.59 ± 0.01 for the free *E. purpurea* gel and 0.60 ± 0.02 for the liposome-loaded gel), suggesting that both formulations maintained comparable internal bonding strength. After 2 months of storage, the gel with free *E. purpurea* showed an increase in firmness (0.54 ± 0.01 N) and springiness (0.87 ± 0.02), surpassing the liposome-loaded gel, which exhibited a slight reduction in firmness (0.50 ± 0.01 N) and springiness (0.80 ± 0.02). Cohesiveness values remained stable for both gels (0.61 ± 0.01 for the free *E. purpurea* gel and 0.60 ± 0.02 for the liposome-loaded gel).

### 2.6. Antioxidant Activity of Gels (DPPH Assay)

The antioxidant capacity of the gels was evaluated using the DPPH assay and quantified in terms of Trolox equivalents (TE, mM/g gel). The gel containing free extract EP exhibited a slightly higher radical scavenging activity (75.15 ± 0.15% inhibition, corresponding to 1.41 ± 0.01 mM TE/g) compared to the EP_L gel (72.69 ± 0.41% inhibition, 1.37 ± 0.01 mM TE/g). As shown in Table 4, both formulations demonstrated good antioxidant activity and similar values suggest that encapsulation preserved the antioxidant properties of EP, supporting the protective effect of liposomes on sensitive phytochemicals.

### 2.7. Cumulative Release and Kinetic Modeling of Polyphenols from Free and Liposome-Encapsulated E. purpurea Gels

**Figure 5:**
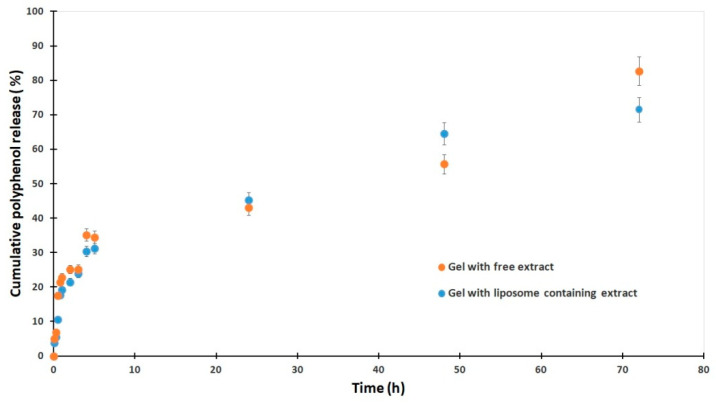
Cumulative release profiles of polyphenols from *E. purpurea* gels: comparison between free extract and liposome-encapsulated formulations over 72 h. Data represent mean ± SD (*n* = 3).

The cumulative release profile of polyphenols from gels containing *E. purpurea* showed distinct differences between formulations with free extract and those with liposome-loaded extract (Figure 5). In the initial phase, the gel with free extract exhibited a slightly more rapid release, reaching 22.8% at 1 h compared to 19.2% from the liposomal gel. After 24 h, release from the free extract gel progressed more slowly, with 43.0% at 24 h, whereas the liposomal formulation surpassed it with 45.2%, indicating enhanced sustained release. Notably, after 48 h, the liposomal gel released 64.5%, markedly higher than the 55.7% from the free extract gel, demonstrating the ability of liposomes to prolong release. However, by 72 h the free extract gel reached a higher cumulative release (82.7%) compared to 71.5% for the liposomal system, suggesting that encapsulation delays complete release but maintains more controlled kinetics over time. Overall, these results confirm that liposomal incorporation reduces the initial burst, enhances sustained release, and modifies the overall release kinetics of polyphenols from the gel matrix. No statistically significant difference was observed between the free and liposomal gels using a two-tailed *t*-test (*p* = 0.101).

To better understand the release mechanisms of polyphenols from dermatocosmetic gels, the experimental data were subjected to kinetic modeling using several mathematical approaches, including zero-order, first-order, Korsmeyer–Peppas, Weibull and Hixson–Crowell equations. Model fitting was carried out with the KinetDS 3 software package. Model performance was evaluated through the correlation coefficient (R^2^), root mean square error (RMSE), and the Akaike information criterion (AIC). An optimal model was defined as having an R^2^ value close to 1, together with lower RMSE and AIC values. The statistical indices for each model are summarized in Table 5.

The mathematical model’s equations used are as follows:

Zero-order model:

(1) $$M_{t}=K_{0}\cdot t$$

First-order model:

(2) $$M_{t}=100\cdot \left(1-e^{-kt}\right)$$

Korsmeyer–Peppas model:

(3) $$M_{t}=K_{p}\cdot t^{n}$$

Weibull model:

(4) Mt=1−exp−tba

Hixson–Crowell model:

(5) $$M_{t}^{\frac{1}{3}}=K_{HC}\cdot t+M_{0}^{\frac{1}{3}}$$

where

*M_t_* is the amount of polyphenols released at time *t*; *K_0_* is the zero-order model constant; *k* is the first-order model constant; *K_p_* is the Korsmeyer–Peppas constant; *n* is an exponential factor; *a* and *b* are Weibull parameters; *M_0_* represents the total initial amount of polyphenols present in the gel before any release occurs (*t* = 0); and *K_HC_* is the Hixson–Crowell release rate constant.

For plain gel, the Weibull model (R^2^ = 0.919, RMSE = 6.32, AIC = 78.08) has the best fit, closely followed by Korsmeyer–Peppas (R^2^ = 0.897, RMSE = 6.64, AIC = 79.24), while for liposomal gel, the Weibull model again is the best (R^2^ = 0.963, RMSE = 3.38, AIC = 63.04), followed by Korsmeyer–Peppas. Weibull fit suggests a time-dependent release with a flexible curve shape. The release exponent at around 0.44–0.49 indicates Fickian diffusion-dominated release. Korsmeyer–Peppas model shows *n* values of approximately 0.36 (Gel with EP) and 0.42 (Gel with EP_L), which are consistent with diffusion-controlled drug release.

Both formulations follow diffusion-controlled release, best described by the Weibull model. Incorporating liposomes significantly improves control and predictability of *E. purpurea* release, likely due to their ability to modulate drug diffusion and retention in the gel.

## 3. Conclusions

This study shows that topical gels incorporating an extract of lipid nanosystems can be considered promising candidates for the dermal delivery of *E. purpurea* extract. Both transferosomes and liposomes demonstrated successful entrapment of the bioactive compounds, with liposomes offering higher entrapment efficiency, while transferosomes exhibit superior size uniformity. These complementary properties underscore their suitability as delivery platforms, allowing for strategic selection based on specific therapeutic goals. The gel formulations showed excellent physical, chemical, and textural stability over a two-month period. Notably, the incorporation of *E. purpurea* in liposomes enhanced the gel’s elasticity and firmness, which are attributes that may improve user experience through better skin feel and spreadability. The antioxidant activity of the incorporated *E. purpurea* remained comparable to that of the free extract, affirming the protective role of the lipid carriers in preserving bioactivity. In vitro release studies confirmed that both systems follow a diffusion-controlled release pattern, which is best described by the Weibull model. The presence of liposomes contributed to a more controlled and predictable release profile, likely improving skin retention and therapeutic efficacy. In conclusion, these results highlight the potential of nanosystem-based topical gels as innovative and effective solutions for modern skincare. However, in vivo and clinical studies—along with scale-up research—are needed to fully confirm their therapeutic potential and practical use in commercial skincare products.

## 4. Materials and Methods

### 4.1. Materials

Standard phenolic compounds used for identification and quantification in *E. purpurea* extract included: protocatechuic acid (Tokyo Chemical Industry, Tokyo, TCI, Japan >98%), caftaric acid (Sigma-Aldrich, Merck Group, Darmstadt, Germany, ≥98%), vanillic acid (TCI, >98%, GC-grade), syringic acid (Molekula GmbH, Munich, Germany, >98.5%), (–)-epicatechin (TCI, >98%), trans-ferulic acid (TCI, >98%), ellagic acid dehydrate (TCI, >98%), rutin hydrate (Sigma-Aldrich, 95%), and cichoric acid (Sigma-Aldrich, ≥95%). The mobile phases and sample preparations used acetonitrile (Riedel-de Haën, Honeywell, Seelze, Germany), ethanol (Riedel-de Haën), and formic acid (Merck Group, Darmstadt, Germany), along with ultrapure water obtained from a Millipore Direct-Q3UV system (Merck Group, Darmstadt, Germany) equipped with a Biopack UF cartridge.

For the obtaining of lipid nanosystems loaded with *E. purpurea* extract, phosphatidylcholine (from egg yolk, Sigma-Aldrich), and sodium cholate (Sigma-Aldrich) were used. Organic solvents, methanol and ethanol, were acquired from Sigma-Aldrich.

For the preparation of the gel formulations, Carbopol 940, glycerin, and sodium hydroxide were purchased from Sigma-Aldrich, vitamin E (α-tocopherol) from TCI, and Eucalyptus essential oil from Fares, Romania.

The antioxidant activity was assessed through the DPPH assay, using 2,2-diphenyl-1-picrylhydrazyl (TCI) prepared in methanol.

For cytotoxicity and cytoprotective assays were used: murine fibroblast cell line (L-929) obtained from ATCC^®^CRL-6364™ (Manassas, VA, USA), penicillin/streptomycin/neomycin solution (PSN), trypsin-ethylene-diamine-tetraacetic-acid (EDTA) solution, Eagle’s minimum essential medium (EMEM), hydrogen peroxide 30% (*w*/*w*) (H_2_O_2_), L-ascorbic acid, fetal bovine serum (FBS)—all acquired from Sigma-Aldrich (Darmstadt, Germany). CellTiter96^®^aqueous non-radioactive cell proliferation assay was purchased from Promega (Madison, WI, USA).

### 4.2. Plant Material

*E. purpurea* leaves were harvested at maturity from Dâmbovița County, Romania (latitude: 45° 18′15.4″ N, longitude: 25°23′28.4″ E) and identified by the Extractive Biotechnologies team of the National Institute for Chemical-Pharmaceutical Research and Development—ICCF, Bucharest, Romania. *E. purpurea* leaves were selected as plant material based on their bioactive phytoconstituents, including polyphenolic compounds, whose cell-protective property against reactive oxygen species has been demonstrated. The extraction technique using 50% ethanol (*v*/*v*) as the solvent to optimize polyphenol recovery was previously described in our earlier publication [44]. The resulting extract was concentrated and standardized based on its total polyphenol content, expressed as gallic acid equivalents (GAE).

### 4.3. HPLC Analysis of Phenolic Compounds

This analysis was performed using high-performance liquid chromatography (HPLC; Shimadzu Nexera 2) equipped with a photodiode array detector (SPD-M30A) (Shimadzu Corporation, Kyoto, Japan) operating over a wavelength range of 250–650 nm, a quaternary pump LC-20ADXR (Shimadzu Corporation), a vacuum degasser DGU-20A5R (Shimadzu Corporation), an autosampler SIL-30AC (Shimadzu Corporation), and a column oven CTO-20AC (Shimadzu Corporation). Chromatograms were processed using LabSolutions Lite LC/GC software (version 5.82, Shimadzu Corporation). Separation was achieved via gradient elution on a Nucleoshell^®^ C18 reversed-phase column (4.6 × 100 mm, 2.7 µm) using two mobile phases: 2.5% aqueous formic acid (mobile phase A) and 90% aqueous acetonitrile with 2.5% formic acid (mobile phase B). The elution program was adapted from Nicoletti et al. [45]: an isocratic elution for 1.5 min with 5% mobile phase B, followed by a 6 min linear gradient to 9% B and another 6 min linear gradient to 13.5% B, then an isocratic elution at 13.5% B for 2.5 min, a 5 min linear gradient to 18.5% B, followed by a 1 min linear gradient to 22.5% B, isocratic elution at 22.5% B for 3.5 min, a 4.5 min linear gradient to 35% B, a 5 min linear gradient to 100% B, and isocratic elution for 5 min with 100% B. Finally, the gradient was returned to the initial concentration, and the column was equilibrated for 10 min before the next injection. The flow rate was set at 0.4 mL/min, separation was carried out at 20 °C, and the injection volume was 1 µL. All solvents used for the mobile phases were previously filtered through 0.45 µm nylon membranes. The chromatogram of *E. purpurea* extract was recorded at 330 nm. Polyphenolic compounds were identified by comparing retention times and the similarity of UV–Vis spectra with those of standard substances. Each HPLC standard was dissolved in ethanol at a concentration of 100 mg/L, and calibration curves for quantifying the phenolic compounds were obtained by injecting five solutions with concentrations ranging from 0.5 to 100 mg/L for each standard.

### 4.4. Total Polyphenol Content and Antioxidant Activity of E. purpurea Extract

The total polyphenol amount of *E. purpurea* extract was measured using the method of Folin–Ciocalteu [46]. The concentration of total polyphenolic compounds was quantified using a gallic acid standard curve (concentration range: 0.01–0.1 mg/mL; calibration equation: y = 0.01332x + 0.0262; R^2^ = 0.99584). Results were expressed as milligrams of gallic acid equivalents per gram of dry extract (mg GAE/g).

Antioxidant activity was evaluated using the DPPH (2,2-diphenyl-1-picrylhydrazyl) radical scavenging assay. 50 µL of extract was mixed with a 2950 µL DPPH methanolic solution (0.025 g/L) and incubated in the dark for 30 min. The absorbance was measured at 517 nm using a UV/VIS spectrophotometer (Jasco V-630, Portland, OR, USA). The antioxidant activity was calculated as the percentage of DPPH radical inhibition. As positive controls, quercetin (0.0001–1 mg/mL) and caffeic acid (0.0001–1 mg/mL) were used. Five replicates were carried out in experiments to evaluate the antioxidant activity.

### 4.5. Preparation of Lipid Nanosystems Loaded with E. purpurea Extract

The lipid nanosystems containing *E. purpurea* extract were prepared following the method described by Pavaloiu et al. [44], which is based on thin-film hydration combined with sonication and extrusion. For the present work, this protocol was slightly modified, mainly by adjusting the concentrations of phosphatidylcholine and sodium cholate, and other formulation parameters, in order to obtain the nanosystems investigated here.

For liposome preparation, phosphatidylcholine was used at 100 mg per 10 mL ethanol. For transferosome preparation, phosphatidylcholine and sodium cholate were used in a 10:2 *w*/*w* ratio (100 mg phosphatidylcholine, 20 mg sodium cholate), dissolved in 10 mL of ethanol. A standardized quantity of *E. purpurea* extract (25 mg) was incorporated into the lipid phase. To ensure phospholipid swelling and consistent extract incorporation, the mixtures were kept at room temperature. Thin, uniform lipid films were obtained by removing the organic solvent at 35 °C under decreased pressure using a rotary evaporator (Laboranta 4000, Heidolph Instruments GmbH & Co. KG, Kelheim, Germany). To help stabilize the vesicles, these films were hydrated with bidistilled water at a temperature higher than the lipid phase-transition point (35 °C). The resultant suspensions were then maintained at 25 °C for two hours. The lipid suspensions were subjected to sonication for 20 min at 50% amplitude (Sonorex-Digital-10P, Bandelin-Electronic, Berlin, Germany) in order to decrease the size of the vesicles and achieve a uniform dispersion. This was followed by seven separate extrusions through polycarbonate membranes with pore sizes of 0.4 µm and 0.2 µm. Centrifugation was used to separate the resulting lipid nanosystems from the unentrapped extract for 20 min at 12,000 rpm and 5 °C. Distilled water was used to properly redistribute the pellet that contained the encapsulated vesicles. The formulations were prepared in triplicate and stored at 4 °C until further analysis.

### 4.6. Characterization of Lipid Nanosystems Loaded with E. purpurea Extract

Particle size, PDI, EE, and release profile were used to describe the nanosystems. Dynamic light scattering (DLS) was used to measure particle size and PDI. To minimize multiple scattering effects, the lipid suspensions were diluted with distilled water at a 1:10 ratio. EE was determined as a ratio between the encapsulated polyphenols in the lipid nanosystem and the initial amount of extract added. The total polyphenol content encapsulated in the lipid nanosystems was measured using the Folin–Ciocalteu method, following the procedure reported in the literature [41]. The stability of the lipid nanosystems loaded with *E. purpurea* was evaluated by monitoring their characteristics over time (3 months of storage at 4 °C).

### 4.7. In Vitro Cytotoxicity Assay

The MTS test was used to assess the cytotoxicity of both free plant extract and its corresponding vesicular formulation on murine fibroblast L929 cells. L-929 fibroblasts were cultured in 25 cm^2^ flasks in EMEM supplemented with 10% FBS and 1% PSN. At approximately 75% confluence (~48 h), cells were detached with trypsin-EDTA, neutralized with fetal bovine serum, pelleted by centrifugation (1200 rpm, 10 min), and resuspended in culture medium at 1 × 10^6^ cells/mL. Cells were seeded in 96-well plates at 7000 cells/well and incubated for 24 h at 37 °C, 5% CO_2_. Cells were exposed to different concentrations of samples (5, 10, 50, and 100 µg/mL) for 24 h at 37 °C, 5% CO_2_. Cells incubated only with medium were used as a negative control. Cell viability was assessed using the CellTiter 96^®^ AQueous Non-Radioactive Cell Proliferation Assay (MTS, Promega Corporation, Madison, WI, USA). After washing twice with serum-free EMEM, 100 µL of MTS reagent (1:10 in medium) was added per well, and plates were incubated for 3 h at 37 °C in the dark. Absorbance was measured at 490 nm using a microplate reader (Chameleon V Plate Reader, LKB Instruments, Victoria, Australia). Viability was expressed as a percentage of untreated controls (100% viability). All samples were sterilized by exposure to ultraviolet light for three hours. The cytotoxicity assay was carried out in triplicate.

### 4.8. Evaluation of Cytoprotective Effect Under Oxidative Stress

The cytoprotective effect of the samples was evaluated using an in vitro oxidative stress challenge model in L-929 fibroblast cultures, with cell viability (MTS assay) as the endpoint. This cell line was selected considering the intended topical application of the final products. The tested samples were lipid nanosystems without extract, free extract, and lipid nanosystems loaded with *E. purpurea*. All assays were performed in triplicate, and results are expressed as mean ± SD. Statistical comparisons were conducted using one-way ANOVA, with *p* < 0.05 considered significant.

All the samples used in these tests were sterilized by exposure to UV light for three hours prior to testing.

#### 4.8.1. Determination of the H_2_O_2_ Concentration Reducing Cell Viability

L-929 fibroblasts were cultured in EMEM supplemented with 10% FBS and seeded in 96-well plates at 1 × 10^5^ cells/mL. Exposure was carried out at a minimum confluence of about 80%, in medium containing 2% fetal bovine serum for 12 h. Untreated cells serve as a control. Cell viability was measured using the MTS assay. The concentrations of H_2_O_2_ tested were 1, 10, 20, 50, and 100 mM.

#### 4.8.2. Evaluation of the Antioxidant Effect of Extract and Extract-Loaded Lipid Nanosystems

Cells were pre-incubated for 1 or 24 h before the oxidative challenge with different concentrations (5, 10 and 25 µg/mL) of the test samples (free extract, empty transferosomes, empty liposomes, transferosomes loaded with extract, liposomes loaded with extract). Oxidative stress was induced by exposure to 50 mM H_2_O_2_ for 4 h. After treatment, cell viability was determined using the MTS assay. Controls included untreated cells and cells exposed only to H_2_O_2_. Cells incubated with ascorbic acid at the same concentrations as the test samples were used as the positive control. All the samples used in these tests were treated with UV light for three hours for sterilization. Oxidative stress induction assay was conducted in triplicate.

### 4.9. Preparation and Characterization of Gels Containing Liposomes Loaded with E. purpurea

#### 4.9.1. Formulation of Gels with Liposomes Loaded with *E. purpurea*

Two gel formulations were prepared to enable a comparative evaluation of the release profile of *E. purpurea* extract: one gel containing the free hydroalcoholic extract, and a second gel incorporating liposomes loaded with the same extract (Table 6). The gel was prepared by first allowing the Carbopol to fully hydrate in distilled water for 24 h. Glycerin was then added, and gelation was induced by the gradual addition of 5% sodium hydroxide solution under constant stirring, until a homogeneous gel was obtained. During this step, the pH of the formulations was adjusted to the range of 5.1–5.3, ensuring suitability for dermal application. Eucalyptus essential oil (*Eucalyptus globulus* L.) and liposomes loaded with *E. purpurea* extract were incorporated afterward, with gentle and continuous mixing. Vitamin E (α-tocopherol acetate) was also added. We used 1.5% hydroalcoholic extract in the conventional gel and 2% liposomes in the liposomal gel to make sure both formulations contained the same amount of *E. purpurea* extract, allowing for a direct and meaningful comparison of their release profiles. The extract content in the liposomal gel was calculated based on the amount of extract per mg of liposome dispersion, ensuring equivalence with the 1.5% hydroalcoholic extract used in the conventional gel.

#### 4.9.2. Characterization of Gels with Liposomes Loaded with *E. purpurea*

Gel formulations were first visually evaluated by placing a small amount on a glass slide and examining it under a 4.5× magnifying lens to assess texture, color uniformity, homogeneity, and fragrance.

The pH of the gels was determined using a calibrated digital pH meter (Mettler Toledo, Columbus, OH, USA) after thorough homogenization. Measurements were taken once the reading stabilized for 30 s, with three readings recorded at different locations; if differences exceeded ± 0.2 units, the measurement was repeated to ensure accuracy.

Texture profiling of the gel was performed using a TX-700 texture analyzer (Lamy Rheology, Champagne-au-Mont-d’Or, France) equipped with a 10 kg force sensor. A two-cycle compression test, simulating product application, was conducted to evaluate firmness (maximum force for initial deformation), cohesiveness (ratio of energy between successive compressions, reflecting structural integrity), and springiness (ability to regain shape after compression). For each test, 40 g of gel was placed in cylindrical containers with leveled surfaces, and a hemispherical probe was used. Compression speed was set at 0.8 mm/s, deformation depth at 10 mm, trigger force at 5 g (0.05 N), with a 5 s interval between cycles. All measurements were conducted at ambient temperature, and each formulation was tested in triplicate to ensure reproducibility.

The stability of the gels was evaluated over a 60-day period, during which samples were stored in amber glass containers at 4 °C. After this period, assessments of visual appearance, pH, and fragrance were conducted to identify any indications of degradation or instability.

#### 4.9.3. Determination of Antioxidant Activity of Liposomally Entrapped EP in Gels

The antioxidant activity of the liposomally entrapped EP was evaluated using the DPPH (2,2-diphenyl-1-picrylhydrazyl) free radical scavenging assay. Approximately 1 g of each gel formulation containing liposomal EP was mixed with 10 mL of ethanol in order to extract the active compounds from the liposomal matrix. The mixtures were vigorously vortexed for 5 min, followed by sonication for 20 min to enhance extraction efficiency. After extraction, the samples were filtered twice to obtain a clear solution. An aliquot of 0.6 mL from the filtered extract was then added to 2.4 mL of a freshly prepared 0.025 g/L DPPH solution in ethanol. The resulting mixture was incubated in the dark at room temperature for 30 min to allow the reaction to occur. The decrease in absorbance at 517 nm was measured using a UV–Vis spectrophotometer (Jasco V-630, Portland, OR, USA). The antioxidant activity was expressed as percentage inhibition of the DPPH radical (%Inhibition, Mean ± SD), as well as in Trolox equivalents (mM/g ± SD), based on a Trolox calibration curve (y = 51.705x + 1.670; R^2^ = 0.9985). This method was used to confirm that the antioxidant activity of EP was preserved after encapsulation in liposomes and incorporation into the gel formulation.

### 4.10. In Vitro Evaluation of Polyphenol Release from Lipid Nanosystems-Loaded Dermatocosmetic Gel

The release behavior of polyphenols from the dermatocosmetic gel containing liposome loaded with *E. purpurea* was studied using a Franz diffusion cell apparatus. 0.5 g of gel was placed in the donor compartment, while the receptor compartment was filled with 100 mL of phosphate-buffered saline (PBS, 0.1 M, pH 7.4) to mimic physiological conditions. The system was maintained at 32 °C to simulate skin temperature, with continuous stirring at 100 rpm to ensure uniform mixing in the receptor phase.

Samples of 1 mL were withdrawn from the receptor medium at predetermined intervals—5, 15, 30, 45, 60 min and 2, 3, 4, 5, 6, 24, 48, and 72 h—and immediately replaced with fresh PBS to maintain sink conditions. Polyphenol concentrations in each sample were measured using UV–Vis spectrophotometry. This approach enabled tracking the cumulative release of polyphenols over time, providing insight into the sustained release potential of the liposome gel compared to the gel containing free extract.

### 4.11. Statistical Analysis

All experiments were performed in triplicate, and results are presented as mean ± standard deviation (SD). Data were analyzed using one-way ANOVA. For the release experiments, pair-wise two-tailed *t*-tests were used to compare the difference between formulations. Differences were considered statistically significant at *p* < 0.05.

## Figures and Tables

**Table 1:** Retention times and concentrations of the compounds identified in the *E. purpurea* extract.

Compound	Retention Time (min)	Concentration (mg_compound_/g_extract_)
Protocatechuic acid	7.01	0.02 ± 0.01
Caftaric acid	10.94	15.42 ± 0.02
Trans-ferulic acid	24.99	0.10 ± 0.01
Rutin hydrate	26.01	0.31 ± 0.01
Chicoric acid	29.22	19.53 ± 0.01

**Table 2:** Physicochemical parameters of transferosomes and liposomes.

Sample	Particle Size (nm)	PDI	EE (%)
Empty transferosomes	102.2 ± 1.1	0.40 ± 0.03	-
EP_T	156.3 ± 1.1	0.08 ± 0.02	63.10 ± 1.03
Empty liposomes	63.2 ± 0.4	0.42 ± 0.02	-
EP_L	199.1 ± 0.2	0.44 ± 0.01	75.15 ± 1.24

EP_T—transferosomes loaded with EP extract, EP_L—liposomes loaded with EP extract.

**Table 3:** Features of gels containing liposomes loaded with *E. purpurea* (EP) and free extract.

Features	Gel with EP	Gel with EP_L
Features after 1 day		
Organoleptic evaluation	Homogeneous, visually transparent, aromatic odor	Homogeneous, visually transparent, aromatic odor
pH	5.15 ± 0.11	5.17 ± 0.10
TPA profile	Firmness (hardness): 0.43 ± 0.02 N Cohesiveness: 0.59 ± 0.01 Springiness: 0.74 ± 0.02	Firmness (hardness): 0.50 ± 0.01 N Cohesiveness: 0.60 ± 0.02 Springiness: 0.85 ± 0.02
Features after 2 months		
Organoleptic evaluation	Homogeneous, visually transparent, aromatic odorNo evidence of phase separation, sediment formation, or changes in texture was observed	Homogeneous, visually transparent, aromatic odor No evidence of phase separation, sediment formation, or changes in texture was observed
pH	5.25 ± 0.02	5.28 ± 0.03
TPA profile	Firmness (hardness): 0.54 ± 0.01 N Cohesiveness: 0.61 ± 0.01 Springiness: 0.87 ± 0.02	Firmness (hardness): 0.50 ± 0.01 N Cohesiveness: 0.60 ± 0.02 Springiness: 0.80 ± 0.02

**Table 4:** Antioxidant activity of gels containing free extract (EP) and liposomes loaded with EP (EP_L).

Sample Code	%Inhibition (Mean ± SD)	Trolox Eq. (mM/g) ± SD
Gel with EP	75.15 ± 0.15%	1.41 ± 0.01
Gel with EP_L	72.69 ± 0.41%	1.37 ± 0.01

**Table 5:** Kinetic modeling parameters for polyphenol release from gels, including R^2^, RMSE, and AIC values for zero-order, first-order, Korsmeyer–Peppas, Weibull, and Hixson–Crowell models.

	Gel with EP			Gel with EP_L		
Model	R^2^	RMSE	AIC	R^2^	RMSE	AIC
Zero order	0.860	7.72	82.87	0.858	7.82	83.19
First order	0.492	10.36	89.93	0.501	13.59	96.44
Korsmeyer–Peppas	0.897	6.64	79.24	0.939	5.74	75.75
Weibull	0.919	6.32	78.08	0.963	3.38	63.04
Hixson–Crowell	0.638	8.69	85.71	0.642	10.34	89.88

**Table 6:** Composition of gels containing free extract and liposomes loaded with EP.

Component	Gel with EP	Gel with EP_L
Carbopol 940	1%	1%
Glycerin	8%	8%
EP	1.5%	-
EP_L	-	2%
Vitamin E	0.1%	0.1%
Eucalyptus essential oil	0.1%	0.1%
Sodium hydroxide 5%	q.s	q.s
Purified water	Up to 100%	Up to 100%

## Data Availability

The original contributions presented in this study are included in the article. Further inquiries can be directed to the corresponding author.

## Supplementary Materials

The following supporting information can be downloaded at https://www.mdpi.com/article/10.3390/gels11100801/s1. Table S1: Reference phenolic compounds: retention time (RT) and standard deviation (SD), maximum absorbance (λ_max_), calibration curve equation (y = ax + b), and correlation coefficient (R^2^).
