# Supplementary material for: Development and Characterization of Topical Gels Containing Lipid Nanosystems Loaded with Echinacea purpurea
Source: Gels. 2025 Oct 5;11(10):801. doi: 10.3390/gels11100801 (PMC12562447; doi:10.3390/gels11100801)
Supplement: Supplementary file 1 [file gels-11-00801-s001.zip › gels-3894673-supplementary.pdf]

Development and Characterization of Topical Gels Containing Lipid Nanosystems Loaded with *Echinacea purpurea*

Ramona-Daniela Pavaloiu [1], Georgeta Neagu [1], Adrian Albulescu [1,2], Mihaela Deaconu [3], Anton-Liviu Petrica [4], Corina Bubueanu [1] and Fawzia Sha'at [1,*]

[1] National Institute for Chemical-Pharmaceutical Research and Development, ICCF, 031299 Bucharest, Romania; pavaloiu.daniella@gmail.com (R.-D.P.); getabios@yahoo.com (G.N.); adrian.albulescu@virology.ro (A.A.); corina.bubueanu@yahoo.com (C.B.)
[2] Department of Molecular Virology, Stefan S. Nicolau Institute of Virology, 030304 Bucharest, Romania
[3] Faculty of Chemical Engineering and Biotechnologies, University Politehnica of Bucharest, 011061 Bucharest, Romania; mihaela_deaconu@yahoo.com
[4] Faculty of Business and Tourism, Bucharest University of Economic Studies, 010374 Bucharest, Romania; petricaanton20@stud.ase.ro
* Correspondence: fawzya.shaat@gmail.com

Table S1. Reference phenolic compounds: retention time (RT) and standard deviation (SD), maximum absorbance (λ_{max}), calibration curve equation (y = ax + b), and correlation coefficient (R^2).

Compound	*RT* ± SD, min	λ_{max}, nm	*a*	*b*	R^2
Protocatechuic acid	7.07 ± 0.09	360	9007.30	-1186.030	0.9997
Caftaric acid	10.96 ± 0.04	329	3741.63	-1004.520	0.9992
Vanillic acid	15.16 ± 0.03	261	8457.44	-1216.690	0.9999
Syringic acid	16.87 ± 0.03	275	6929.03	-667.267	0.9997
(-)-Epicatechin	17.81 ± 0.04	278	1585.50	-273.149	0.9998
Trans-ferulic acid	25.47 ± 0.05	323	3135.95	-769.688	0.9999
Ellagic acid dihydrate	25.62 ± 0.06	252	5128.87	-577.181	0.9996
Rutin hydrate	25.97 ± 0.02	355	3813.00	-838.900	0.9985
Chicoric acid	29.19 ± 0.05	330	10560.60	-1939.030	0.9975
